# Consumer Choices and Habits Related to Coffee Consumption by Poles

**DOI:** 10.3390/ijerph18083948

**Published:** 2021-04-09

**Authors:** Ewa Czarniecka-Skubina, Marlena Pielak, Piotr Sałek, Renata Korzeniowska-Ginter, Tomasz Owczarek

**Affiliations:** 1 Department of Food Gastronomy and Food Hygiene, Institute of Human Nutrition Sciences, Warsaw University of Life Sciences (WULS), Str. Nowoursynowska 166, 02-787 Warsaw, Poland; marlena_pielak@sggw.edu.pl (M.P.); piotr_salek@sggw.edu.pl (P.S.); 2 Department of Quality Management, Gdynia Maritime University, Str. Morska 81-87, 81-225 Gdynia, Poland; r.ginter@wznj.umg.edu.pl; 3 Department of Management and Economics, Gdynia Maritime University, Str. Morska 81-87, 81-225 Gdynia, Poland; t.owczarek@wznj.umg.edu.pl

**Keywords:** coffee, consumer habits, coffee brewing, Poles

## Abstract

Coffee is one of the most popular drinks consumed in the world, also in Poland. In the literature, much attention is paid to the influence of coffee on human health, especially daily intake of caffeine, and also purchasing consumer behavior. There is a lack of research devoted to consumer choices and habits in relation to coffee consumption and brewing method. Therefore, the aim of this study is to describe the characteristics of coffee consumers and present their segmentation based on consumer choices and habits towards coffee consumption. The study was performed using the computer-assisted web interviewing (CAWI) method on a group of 1500 adults respondents in Poland reporting the consumption of coffee. We collected information about consumer choices and habits related to coffee consumption, including brewing method, place of consuming coffee, and factors determining coffee choices. Using cluster analysis, we identified three main groups of coffee consumers. There are “Neutral coffee drinkers”, “Ad hoc coffee drinkers”, and “Non-specific coffee drinkers”. The respondents in the study are not coffee gourmets; they like and consume coffee, but these are often changing choices. To conclude, it can be stated that the Polish coffee consumer prefers conventional methods of brewing coffee (like a “traditionalist”) but is open to novelties and new sensory experiences. Based on study results it is possible to know the coffee drinking habits in Poland.

## 1. Introduction

Coffee is the second most traded commodity in the world. In 2017–2018, the global production of coffee beans from around 60 countries reached approximately 9513 million tones, and achieved USD 200 billion annually [1,2]. In 2018–2019, the consumption of coffee beans was over 165 million 60-kg packages [3]. It is estimated that 500 billion cups of coffee are consumed every day [4]. According to available sources, the highest coffee consumption in Europe is in Scandinavia at approximately 10 kg of coffee per capita per year. In Finland is 12 kg per capita per year; in Norway, 9.9 kg; in Iceland, 9 kg; in Denmark, 8.7 kg, and in Sweden, 8.2 kg. In other European countries, coffee consumption is lower, in Netherlands—8.4 kg, Switzerland—7.9 kg, Belgium—6.8 kg, and Luxembourg—6.5 kg [1]. In Poland, coffee consumption is an average of 2.2–3 kg per capita per year [5].

Many studies [6,7,8,9,10,11] concentrate on the effects of coffee on the body and health, especially disease risk, and daily intake of caffeine with coffee [12,13,14]. Coffee, apart from the unique, characteristic taste and aroma, contains caffeine, and several antioxidants, including chlorogenic acid, lignan, melanoids, cafestrol, trigonelline, and kahweol, which may show a protective effect at the cellular level. The ingredients contained in the coffee infusion are responsible for many beneficial processes that take place in the human body [14,15,16,17,18,19,20,21,22,23,24,25,26,27,28,29,30,31,32,33,34]. Excessive, long-term consumption of caffeine (above 500–600 mg daily) may lead to addiction and many negative symptoms from the body but also show that coffee becomes a risk factor for various diseases while consume above five number of cups per day [35,36,37,38,39,40,41,42,43].

Many studies [44,45,46,47,48,49] focus on the coffee markets, including purchasing consumer behavior and quantified consumption, as well as sustainable development and ethical consumption in line with the principles of fair trade. Few data [47,50,51,52,53,54,55] concern consumption motives, consumer behavior, and preferences as to the method of coffee brewing or aspects related to the proper preparation of the infusion. Coffee is considered a high-quality food. In the minds of consumers, consumption of coffee is more and more often equated with pleasure and positive experiences and is related to lifestyle and social status. Various studies confirm that drinking a cup of coffee is associated with a personal moment of pleasure for the consumer [56,57,58], and characterize coffee as: pleasure, health, and sustainable development [59].

The mentioned previous studies concern on habits, changes in consumer behavior and preferences related to coffee, marketing aspects, and also related to the impact on health, without addressing “technological” issues connected with preparing and consume coffee infusions. The topic of coffee research is related to the cultural and geographic context in which it is conducted [52,53,54,55,60]. In Western economies, a lot of emphasis is placed on issues of sustainable development, including fair trade. In Asia, an important aspect is mainly the behavior and preferences of consumers in relation to café brands or consumption of coffee products [61]. Consumer habits related to choose the coffee brew methods are also changing with technological development. Studies among European consumers [62] indicate that they use different coffee preparation methods. Italians, Swiss, and Portuguese use espresso machines: fully automated, capsule, and drip coffee maker. While Germans mainly use a filter coffee makers to brew coffee. In turn, in the South Africa foodservice market espresso based coffee dominate [63].

Coffee drinking habits, methods of coffee infusion, coffee amount consumed, and additive use in coffee are differentiated between countries and population [57,64,65,66,67,68,69]. Although much research on coffee has been published, there is still a lack of research related to the technological practices of coffee preparation. The available results do not cover the topic in such a comprehensive way as our research, and they refer to the topic in a different context. This study fills this research gap. Coffee is one of the most popular drinks in Poland. Over 80% of adult Poles consume coffee regularly and 60% of adult Poles drink it every day [70,71]. Its price is relatively low, from USD 1.65 (PLN 6.09) per 250 g in 2010, rising to about USD 1.86 (PLN 7.54) for 250 g in 2019 [72]. Drinking coffee is becoming a lifestyle. It is also encouraged by the changing coffee market in Poland, where there is an increasing prevalence of cafe chains. The largest coffee shop chains, such as Tchibo, McCafé, Starbucks, and Costa Coffee, are increasing the number of their outlets all over Europe, including in Poland [1].

The Polish coffee market is worth about PLN 6 billion per year, of which about half is household expenditure [73], which indicates buying coffee by consumers for home preparation. Polish consumers buy coffee beans more often (30% of households). At the same time, their interest in instant coffee is decreasing—in 2014–2019 by 13% in terms of value and by 14% in terms of quantity. This is likely due to the increase in the purchasing of domestic coffee machines. In terms of the sale of coffee machines in the first half of 2020, Poles became the fourth market in Europe in terms of value, after Germany, France, and the Netherlands. A significant proportion (60.8%) of the coffee machines purchased in Poland are automatic machines, which in 2020 accounted for 92% of the value of the whole coffee machine market [74]. The consequence of this was an increase in the coffee bean segment by 28.4% [75]. The market for coffee capsule machines, and thus the demand for coffee capsules, is also growing [76]. The COVID-19 pandemic may also have contributed to the increase in the sales of automatic coffee machines, and thus the increase of demand for coffee beans in 2020.

Therefore, this research aimed to analyze Polish consumer habits towards coffee consumption and their choices connected with coffee, as well as factors influence on choose kind, brand, and method of preparation of the coffee infusion. The second aim of this study was to identify, describe, and compare consumer segments based on differences in individual choices and habits related to coffee consumption.

## 2. Materials and Methods

### 2.1. Questionnaire

The questionnaire structure is presented in Table 1. The questionnaire consists of two parts, of which the first part consists of 14 questions relating to coffee consumption and consumer habits with coffee. The questions concern consumer preferences, decisive factors for purchasing, the frequency of coffee consumption, and the method of preparing coffee infusions. The second part of the questionnaire relates to the respondent’s sociodemographic details—gender, age, education, dwelling place. The questionnaire was designed based on the literature. Questions in questionnaire were based on previous studies: Q1, Q2, Q4, Q12, Q13, Q14 [50]; Q1, Q3, Q5, Q6, Q14 [51]; Q6, Q11, Q12, Q13 [77]; Q2, Q11 [78]; Q1, Q13 [79]; and Q7, Q8, Q9, Q10 [80,81].

The questionnaire was assessed by determining its repeatability. The reliability of the questionnaire was validated using its internal consistency. Cronbach’s alpha test was used to measure internal consistency and reliability. Cronbach alpha coefficient was above 0.74, which indicated acceptable internal consistency. Therefore, the questionnaire and scale used is valid.

### 2.2. Data Collection

The Computer-Assisted Web-based Interviewing (CAWI) method was used to collect all data. The survey was conducted on a group of 1500 adult respondents in Poland that reported the consumption of coffee.

Inclusion criteria of respondents for study were as follows:

1. Each respondent in age between 18 to 65 years old of who agreed to participate in the survey was invited to complete the questionnaire.
2. Everyone consuming the coffee.

The exclusion criterion of respondents was people who do not consume coffee.

This paper were designed as a study with a convenience sampling.

The respondents completed an online questionnaire. A link to the questionnaire in Polish language Google Forms format was sent via Facebook, WhatsApp^®^, e-mail, and students forum. A questionnaire provided on a webpage increases the sense of anonymity and gives an opportunity to participate in the study at a time convenient for the respondent, and in time of pandemic COVID-19 was very useful.

The questionnaire was validated by means of a pilot study with 20 people. All problems were identified, for example, unintelligible questions and questionnaire construction, as well as the lack of response request, which can lead to omitted answer for some questions. Then the questionnaire was completed and amended.

It was estimated on a pilot test that it would take each participant around 10–12 min to complete the form. Each adult respondent who agreed to take part in the study was invited to fill in the questionnaire. The respondents were free to participate in the research. Because the research was non-invasive and the details of the participants remained undisclosed, the research does not fall within the remit of the Helsinki Declaration.

### 2.3. Characteristics of Respondents

The characteristics of the respondents are presented in Table 2. The study involved mainly women, with secondary or higher education, living in different types of dwelling places. The respondents were in the range of 18–65 years old, who had access to a computer, the Internet, and had computer literacy skills.

### 2.4. Data Analysis

The statistical analysis of the results was performed using Statistica software (version 13.3 PL; StatSoft Inc., StatSoft, Krakow, Poland). The ANOVA test was used. Significance of differences between the values was determined at a significance level of *p* < 0.05.

A multi-dimensional cluster analysis calculation was performed to coffee consumer classifications. Segmentation was performed using the hierarchical (connectivity-based) clustering. Specifically, the agglomeration clustering method and *k*-means clustering method were used. The analysis was aimed at creating groups of respondents with a homogeneous approach to the purchase and consumption of coffee.

The measure of similarity used in cluster analysis is the distance in a multidimensional coordinate system. This distance can be defined in many different ways. All the variables are therefore categorical, most on the nominal scale and some on the ordinal scale. For this reason, the analysis uses a measure called percent discrepancy, which is the quotient of the number of dimensions with inconsistent values and the number of all dimensions. When studying distances between clusters of multiple elements, it is also necessary to establish a method for calculating the distances of clusters. The analysis used the complete linkage method, also known as the farthest neighborhood method. The distance between clusters is the distance of the farthest elements of both clusters. After separating the clusters, it was examined whether they really differentiate the studied group. For this purpose, the Analysis of Variance (ANOVA) test with the significance level *p* < 0.05 was used. For all clusters, the means and medians were calculated for all variables [82,83].

The analysis consisted of three stages. In the first stage, the system of variables (questions) was reduced. Using the agglomeration method, variables with similar values were combined into clusters, and then all questions were removed from this cluster, leaving one representative. This allows to eliminate from the study questions that are highly correlated and carry the same information, without losing overall information. In the second stage, clusters of cases (respondents) were built using the agglomeration method. The purpose of this stage is to determine the optimal number of clusters. Due to the extremely difficult interpretation of the obtained results, it was decided to create as few reasonable clusters as possible. In the third stage, the elements were finally assigned to clusters using the *k*-means clustering method and the properties of the obtained communities were analyzed. Due to the large number of numerical values, the article does not present detailed values of the measures and test statistics used, but only the conclusions obtained from them, confirmed by graphs.

## 3. Results

### 3.1. Type of Coffee Consumed by Respondents

Respondents primarily choose instant coffee (50.9% of respondents), ground roasted coffee (45.9%), and roasted coffee beans (37%). A significant percentage of respondents also choose grain coffee (17.7%), coffee beverages (13.1%), and flavored coffee (11.5%). Few people reported a consumption of decaffeinated coffee (7.7%) or low-acid coffee (1.3%).

The choice of the type of coffee correlated with age, education, and dwelling place (*p* < 0.05). Roasted coffee beans were significantly more often chosen by people aged 18–25 years, with higher education, and living in cities with above 100,000 inhabitants, while ground roasted coffee was chosen by people aged 31–40 years. Young people (18–25 years of age) significantly more often drink flavored coffee and coffee beverages than others group of consumer. People aged 25–30 years and inhabitants of rural areas consumed grain coffee significantly more often. In turn off, people aged 51–65, with secondary education, and living in cities up to 100,000 inhabitants more often drink instant coffee.

Among the coffee brands, the respondents most often chose Jacobs (44% of indications), Nescafe (36.8%), Tchibo (30.9%), MKCafe (34.9%), and Lavazza (28.4%). Less frequently mentioned were Maxwell House (8.7%), Prima (8.1%), Segafredo (6.8%), and Pedro’s (6.1%). Other brands were mentioned by less than 1% of the respondents. The large variety of coffee brands on the market means that everyone will find something for themselves, and the choice of the brand depends on consumer preferences.

### 3.2. The Frequency and Place of Coffee Consumption

**Figure 1:**
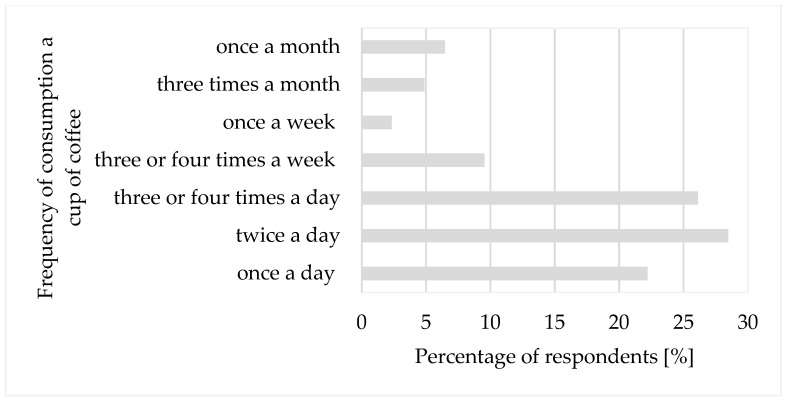
Frequency of consumption coffee by respondents.

All the participants in the study reported drinking coffee. The majority of respondents (76.8%) consume coffee daily, either once, twice, or several times a day (Figure 1). A smaller percentage of respondents drink coffee once or three or four times a week or less. The frequency of coffee consumption was associated with age (*p* = 0.000) and education (*p* = 0.000). However, it did not correlate to gender (*p* = 0.517) or type of dwelling place (*p* = 0.151). People aged 26–50 and people with higher education consume coffee significantly more often—twice to three or more times a day. People aged 51–65 years significantly more often reported coffee consumption once a day, and people aged 18–25 years significantly more often declared coffee consumption once a week or rarely.

The respondents most often drink coffee at home (95.5%) and at work (79.7%), Table 3. The choice of place to drink coffee mainly correlated with gender, age, and dwelling place. In the canteen, coffee was consumed significantly more often by people aged 18–30 years old and living in large cities above 100,000 inhabitants. Women, people aged 18–30 years, with higher education, living in big cities, significantly more often chosen other catering establishments (cafés) to consume coffee. Young people up to 30 years, women and people with higher education significantly more often drink coffee at work and with friends.

### 3.3. Factors Affecting Coffee Purchasing

The most important factors (Table 4) affecting the purchasing of coffee included quality and flavor (taste and aroma) of the coffee, as well as habits of consumers (median 5.5–6). Less important factors for the respondents are coffee price, brand, friends’ opinions, and the features of coffee such as origin, acidity, strength, or degree of roasted (median 5). The least important factors for the respondents were packaging, presentation on the shelf in the store, promotion, advertising, convenience, and coffee health aspects (median 4). The smallest differentiation of respondents’ assessments was obtained in the Convenience and Promotion factors. These factors were assessed as insignificant. Respondents do not pay attention to these factors. While the greatest differentiation of ratings was obtained for the Flavor (taste and aroma) factor. Respondents also assessed this factor as important from the point of view of purchasing coffee. For many people, this factor is extremely important, but some respondents do not pay much attention to it.

### 3.4. Preparation Methods and Types of Coffee Drunk by Respondents

Almost half of the respondents (*n* = 666, 44.4%) reported that the way of coffee prepare is important for them, while a significant percentage of the respondents (*n* = 391, 26.1%) only sometimes paid attention on brewing methods. For others (*n* = 443, 29.5%), is the preparation method was not important.

The respondents like different methods of brewing and different of coffee beverages (Table 5). The most frequent method of brewing coffee stated was coffee made with boiling water in a cup or glass (89.7%), followed by preparing in a pressure coffee machine (77.7%) and in a drip coffee maker (61.5%). The most popular types of coffee were espresso (90.6% of indications) and cappuccino (84.1%), latte or latte macchiato, and frozen coffee (approximately 75% respectively). Americano was reported by 51.4% of the respondents. Other methods, such as lungo, flat white, frappé, with alcohol, doppio, café au lait, frappuccino, café Corto, brewing methods without an espresso machine, and Viennese coffee, were mentioned by less than 10% of respondents.

As an addition to their coffee infusion, most respondents choose milk (69.6%), while fewer choose cream (17.7%). A significant percentage (43.7%) of the respondents sweeten their coffee with sugar. Few of the respondents (7.7%) use sugar substitutes (sweeteners). Almost 40% of respondents drink coffee without additions. Other additions such as cinnamon, cocoa, chocolate, cardamom, syrup, or ice cream were used by 8.8% of respondents.

People aged 31–40 years, with higher education, living in large cities (>100,000 inhabitants) drink coffee without any additions significantly more often (*p* < 0.05). Coffee is more likely to be drunk with sugar by men (*p* = 0.0025), people aged up to 25–30 or 51–65 years, people with vocational education, and people living in cities with less than 10,000 inhabitants. Coffee with milk is more likely to be drunk by women (*p* = 0.00001), people aged 31–40 years (*p* = 0.00001), people with higher education (*p* = 0.005), and people living in both small and large cities. Cream is mostly added by people aged 51–65 years, and people living in cities with 50,000–100,000 inhabitants (*p* < 0.05).

According to the respondents, the most important factors in a coffee infusion are flavor (*n* = 1358, 90.5%) and aroma (*n* = 1086, 72.4%). Coffee appearance (*n* = 254, 16.9%) and color (*n* = 264, 17.6%) are less important.

For the preparation of coffee, the respondents mainly use tap water (64.7%), using 1–2.5 teaspoons of ground coffee (54.3%) or using a coffee machine measuring cup or capsules (13.8%) to measure the amount of coffee. The majority of respondents (80%) did not know the coffee brewing temperature, which should be lower than 98 °C, although this information is given on every coffee package. Over 50% of the respondents did not pay attention to the brewing time (Table 6). Thus, the study participants were not “experts” in the field of coffee brewing.

### 3.5. Characteristics of the Respondents in Terms of Choices and Habits Related to Coffee Consumption

In order to reduce the number of variables present in the study, the agglomeration method of cluster analysis was used. The role of this method is to create groups of questions with very similar answers. This eliminates the variables which carry the same information, and thus attempts to simplify and facilitate inference. The removal of these variables from the study at the same time does not cause a significant loss of the information that was obtained as a result of the survey. In the agglomeration method, percent discrepancy and full bond were used as the distance measure.

**Figure 2:**
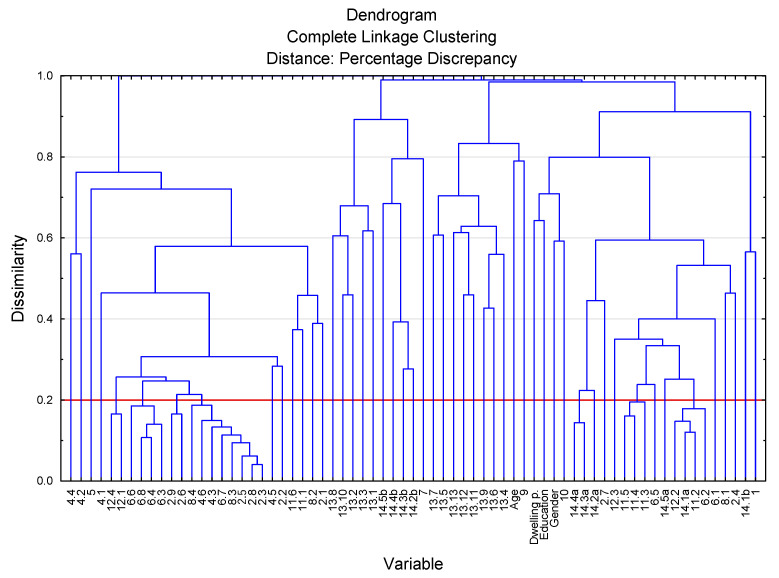
Chart agglomeration of variables.

The agglomeration of variables for a bond distance smaller than 0.2 was adopted as the limit (over 80% of concordant responses). As a result, the variables in seven branches were reduced (Figure 2). The variables in each branch are very closely related, and you can replace them with one variable that represents them. The result of this operation was the reduction of the number of variables by 18, with no significant loss of information carried by them. Fifty-one variables were left for further analysis.

**Figure 3:**
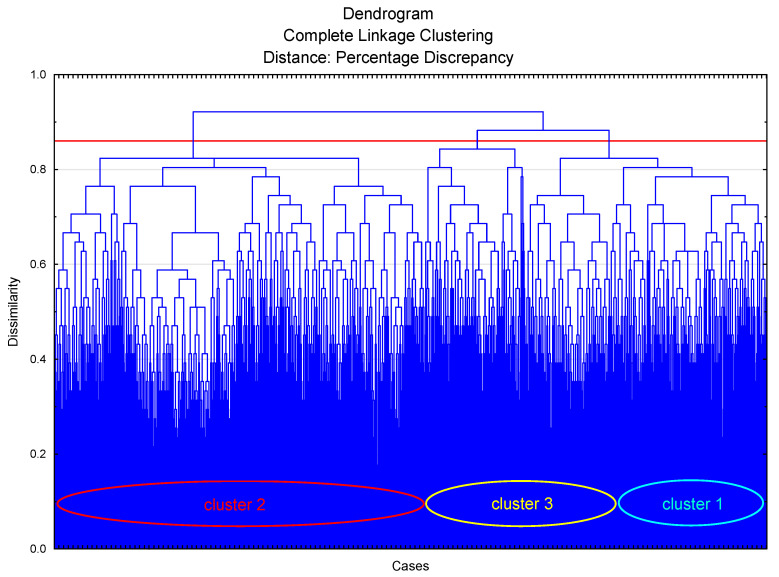
Chart agglomeration of cases.

In the second stage of the analysis, the aim was to create the smallest possible number of groups of cases (respondents) behaving in a similar way to each other. For this purpose, the agglomeration method was used in the cluster analysis with the same assumptions as for the reduction of variables. The agglomeration results are presented in Figure 3.

In the agglomeration, the binding distance of 0.86 was assumed as the cut-off level (the red line in the diagram). The adopted cut-off value made it possible to distinguish three groups of respondents. The confirmation of the validity of the selection of such a cut-off level is the bond distance diagram. The distance of the mates for which the plot becomes the most vertical is taken as the cut-off level. This indicates large distances between successive agglomerations and suggests the emergence of natural case groups.

**Figure 4:**
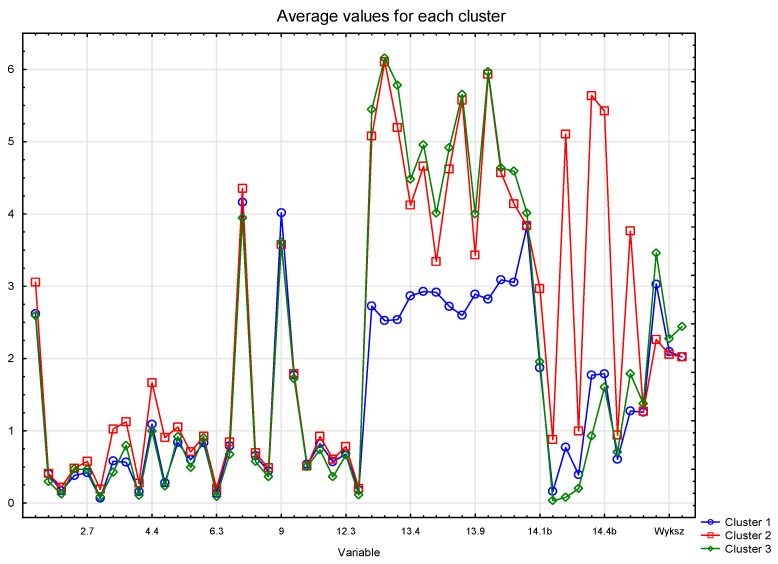
Average values for all variables in three clusters.

The agglomeration method made it possible to determine the optimal number of respondent groups. However, for the precise assignment of individual cases to each of the three groups, further analysis of these groups was performed using the k-means method of cluster analysis. The clusters of the following numbers were obtained: clusters of 1–295 cases, clusters of 2–709 cases, and clusters of 3–496 cases. The results of the analysis of variance performed for all the variables confirm the validity of the division performed. For almost all variables (except two), the proposed division significantly differentiates the community in a statistically significant manner. In other words, the mean values of almost all variables are significantly different in the three proposed groups of respondents. The values of these averages and the relations between them are shown in Figure 4.

Due to the fact that the variables are not quantitative, the obtained mean results cannot be interpreted in terms of their value, but are only an indication of the relationship between the mean values. They make it possible to assess how often the values of variables appear in one cluster compared to another cluster.

On the basis of the diagram of means, it can be concluded that for Questions 1 to 12.4, the respondents in all groups gave similar, but statistically significantly different, answers. It can be seen that the means for Cluster 2 usually have the highest values, and the means for Cluster 3 have the lowest values. Much larger differences are visible in the case of the answers to Questions 13 and 14.

Based on the cluster analysis, the profiles of preferences of coffee consumers were determined. Three profiles were identified:

1. “Neutral coffee drinkers”—Cluster 1,
2. “Ad hoc coffee drinkers”—Cluster 2,
3. “Non-specific coffee drinkers”—Cluster 3.

When buying coffee, the respondents belonging to Cluster 1 (*n* = 295) were clearly less influenced than others by the factors indicated in Question 13. Taking into account that in the case of the remaining questions their average answers were usually between the answers of the other respondents, they can be characterized as people with a neutral or even indifferent attitude towards coffee. They can be characterized as “coffee drinkers” of habit: they like to drink coffee, but they do not mind what type. Representatives of this group are women, people aged 31–40 years, people with higher education, and people living in cities of 50,000–100,000 inhabitants. When buying coffee, these consumers do not pay attention to the information on the packaging, the opinions of friends, presentation on the shelf, or advertising. They drink the strongest coffee and often drink coffee from an espresso machine; quite often they drink coffee outside the home, with friends, and in canteens.

The respondents from Cluster 2 (*n* = 709) behaved differently. Their answers to questions from the group of 14 questions indicate a much lower frequency of coffee consumption than others. Coffee is drunk outside the home much more often than other people. Most of the questions (except Question 13) were answered on average with the highest value. It can be stated that they drink coffee sporadically and at the same time are more likely to consume different types of coffee and prepared in more different ways than others. This would indicate a randomness in coffee consumption: they drink coffee, but without preferences to brewing method and type of coffee. They are young consumers, not connoisseurs, who drink coffee as part of their lifestyle. This group is mainly represented by women, people aged 25–30 years, people with higher education, and people living in large cities (over 100,000 inhabitants).

Cluster 3 (*n* = 496) respondents constitute the rest of the respondents and cannot be characterized in any unequivocal way. The average representative of Cluster 3 are women, people aged 41–50, people with secondary education, and people living in cities with an average size of 10–50,000 inhabitants. When buying coffee, they take into account factors similar to the ones considered by Cluster 2 consumers, and they take them into account to an average extent. However, these consumers rarely drink coffee outside their homes.

Concluding, Polish consumers do not show clear preferences as to the choice of coffee and are not yet “specialists” in the field of coffee brewing, as evidenced by the answers to individual questions, especially when it comes to brewing methods. It seems that they are experimenting in this regard for the time being, choosing a considerable variety of coffees. However, coffee is becoming an integral part of social life, especially among young people.

## 4. Discussion

### 4.1. Consumer Coffee Choices

The respondents indicated quality, flavor, habits, brand, and price as the most important factors affecting the purchase of coffee. Other authors also highlighted these factors [51,84,85]. Numerous studies confirm that the main motive for drinking coffee, and thus the main factor for the purchase of coffee by consumers, is its flavor and aroma, and the feeling of pleasure when consuming it [50,52,57,68,77,86,87,88,89,90], as well as the atmosphere in which coffee is consumed and the emotions that accompany the consumer while drinking it [77]. Other factors include social recognition of the value of coffee and its stimulating “magic effect”, as well as its physical impact on the body, e.g., the ability to aid digestion or increase blood pressure. The direct factors affecting the purchase of coffee are the price and the quality/price ratio, reported flavor and aroma, infusion intensity, and, above all, buying habits [77,91]. It is worth mentioning that consumers are also interested in buying coffee with “health claims” [77].

Other studies, such as this, confirm that one of the factors in consumers purchasing is coffee habits, and family traditions, which can then influence the place of consumption and the type of coffee consumed. Coffee consumption behavior depends on culture and traditions, especially coffee drunk at home. Culture and traditions are also a source of knowledge and information, and creates behavior related to coffee consumption [56,57,60,64,77]. Samoggia and Riedel [77] report that consumers for whom flavor, pleasure, tradition, and habits are the main factor in purchasing and reason for drinking coffee do not consider its beneficial effects on health. On the other hand, if they make a purchase without accompanying emotions, they are more likely to discover new product [77].

In the selection of coffee brands available on the market, the respondents in this study chose typical brands known on the European market—Tchibo, Jacobs, Lavazza, Nescafe, MKCafe, and Maxwell House—which is probably related to their greater promotion and advertising, which affects customers, although respondents did not indicate this factor as decisive for the purchase of coffee. This confirms the results obtained by other authors [51].

The results imply that young respondents significantly more often choose speciality coffee. Similar results indicate Lewin et al. [92], but van der Merwe and Maree [63] found that is no significant relationship between age and speciality coffee consumption. Only a few respondents in this study choose decaffeinated coffee, similar like other authors [60].

### 4.2. Respondents’ Habits Related to Coffee Consumption

The results we obtained regarding the place of coffee consumption from the respondents are consistent with other data from Poland [51,74], and in other countries like Denmark, Sweden, Norway, UK, France, Greece, Spain, and Italy [60]. Home and work are the most popular places to drink coffee. A significant percentage of people participating in this study also mentioned cafés (61.5%) and canteens (45.8%). Such a high proportion of coffee consumption in catering establishments is probably related to the specifics of our research group, which included people aged 18–65 years who were professionally active or studying. Such people are associated with a more mobile lifestyle, possibility of drinking coffee in a café or for a social occasion, and also are more likely to drink their first coffee at home and another one at the workplace. The data from the “Poland on a plate” report [93] confirm this findings, while other data indicates that only 5–6% of Poles drink coffee in catering establishments [51,74]. According to Euromonitor International Coffee [61], the leading factors driving the growth of the coffee market are innovation in the field of consumption outside the home. The other authors [56] report that consumers who drink coffee in cafés associate coffee with the attributes of happiness and joy, as well as companionship and stable interpersonal relationships.

Coffee drinking at home is an intimate, private activity, ensuring personal comfort and the opportunity enjoy the experience [56]. The preferences of coffee consumption, both at home and outside, being related to age and social status, are also indicated by other authors [66,67,94,95]. People under 35 years of age are more likely to drink coffee in a café, while middle-aged and older people (>65 years old) drink coffee at home [94,95] or at work [67]. The reason for drinking coffee during a break at work may be the desire to improve mental and physical fitness (functional benefits of drinking coffee), as well as establishing social contacts with colleagues from work (consumption behavior facilitating social integration) [68].

Findings reveal that, 76.8% of respondents drink coffee every day: once, twice, or several times a day. People aged 51–65 years consume coffee once a day. Other authors [51,63] point to a similar relationship. Elderly people usually limit daily coffee consumption probably due to their health [94,95,96].

### 4.3. Methods of Preparing and Serving Coffee Chosen by Respondents

Finding a relationship between the preferred types of coffee or preparation methods is difficult as they may be dependent on the traditions, culture, and customs of each country [57].

More than half (50.9%) of the respondents choose easy-to-prepare instant coffee. Most people (90.6%) reported drinking espresso. Consumers also willingly to choose cappuccino (84.1%) and iced coffee (76%). This is related to the way coffee is prepared by the respondents. Results reveal that, the respondents choose coffee made with hot water in a cup (89%), coffee from an espresso machine (77%), and coffee from a drip coffee maker (61%). The popularity of pressure brewing method [97], and methods without the use of an espresso machine, i.e., alternative brewing methods are also indicated by other authors [98]. Consumers choose the easy, quick way of preparing coffee, as evidenced by the increase in sales of capsule coffee machines, which can also be observed in Poland. They have attracted the interest of consumers thanks to ease of use and convenience, including easy dosing, as well as the low prices of the coffee machine [66,99].

In Poland, new trends have also been identified in the preparation of cold brew coffee, which is drunk by nearly 13% of respondents. The trend is becoming popular worldwide, as is the interest in consuming coffee outside the home and reducing caffè mocha consumption [94,95].

Findings reveal that, for over half of the respondents (55.6%), the method of brewing is not important, although this is an important stage in the preparation of coffee. Pre-infusion, also known as “blooming”, takes place within the first 30 s after pouring a small amount of water onto the ground coffee beans [80]. The duration of coffee brewing and the ratio of coffee to water depend on the brewing method and machine [100].

The sensory quality of coffee infusions, especially creating aroma, is influenced by many factors, the time passed since roasting of the beans [80,101]. An important aspect in the coffee preparation process is brewing, including use the water, which has an optimum pH of 7.0 (the acceptable range of pH is 6.5–7.5). In this study, 64% of respondents use tap water to make coffee. It is worth mentioning that the water pH value affects the coffee taste [80]. In order to improve water quality, filters can be used to reduce water hardness and remove chlorine and organic pollutants, but only 43% of our respondents use filtered water for brewing coffee.

In order to obtain a high sensory quality in coffee, the water temperature for brewing should be 91–96 °C [81]. Immersing coffee in boiling water may lead to bitter infusions [80]. Almost half (49%) of our study participants prepare coffee in this way, and 2% do not pay attention to water temperature, which may also be associated with irregularities in this regard. Coffee infusions prepared in the temperature range of 88–93 °C are characterized by a balanced astringency and bitter taste, appropriate “crema” color, and well-balanced aroma intensity. The infusion that is prepared is also characterized by appropriate density and taste, as well as a sufficiently high concentration of caffeine [102]. A temperature of water below or above than mentioned, effect on coffee infusion quality [103,104,105,106,107]. Some of the new methods of brewing coffee are performed at temperatures below 25 °C and this methods require a longer extraction time [108].

Coffee can be drunk on its own or with milk, sugar, condensed milk, and others additives. The preparation method and additives affect consumer coffee choices [109]. Respondents added different additives to coffee. Almost half of respondents drunk coffee with sugar, and about 70% drunk coffee with milk. According to Landais et al. [60] coffee and tea have a high contribution to sugar daily intake.

### 4.4. Characteristic of Coffee Consumers Based on This Study

Based on the results, it can be concluded that Polish consumers do not display the characteristics of coffee connoisseurs, but are rather experimenting with coffee. They do not have clear preferences regarding the choice of coffee or specialist knowledge of coffee preparation. It should be emphasized that greater knowledge leads to preferences for different types of coffee [110].

Polish consumers consume coffee more often at work than in other places outside home, driven by the stimulating benefits of drinking coffee—improving mental and physical fitness, the opportunity to take a break, and the social aspect. Interestingly, habits can be the key factors influencing coffee consumption—where it is consumed, types of coffee, preparation methods, which is related to the cultural context and traditions of consumers. A country’s traditions and culture can influence both the occasion and the location of coffee drinking. In countries where a tradition of coffee consumption has developed, such as Italy, Brazil, or the USA, the habits of coffee consumption will be different than in Poland.

#### Limitations

There are some study limitations. The results come from a convenient sample, focused on Poles. The study did not include the group of people over 65 years of age, who in Poland usually do not have computer access or Internet access, or have low computer literacy skills. For this group, access via the Internet is more difficult and it is harder to collect data. At the same time, coffee consumption in Poland is the greatest in the over-65 age group. Another limitation is that the consumers of coffee were from only one country.

## 5. Conclusions

The results of the conducted study suggest that the main factors influencing coffee consumption are sensory quality (flavor and aroma), functional (stimulating) motives, habit factors, and socialization motives.

Polish consumers choose coffee because they like its flavor and the pleasure they experience while consuming it. They also drink coffee because of its functional benefits, wanting to enjoy the energizing effects. The least important factors influencing the choice of coffee by consumers are packaging, in-store displays, advertising, and health aspects. Failure by consumers to pay attention to the information on the packaging may result in a lack of knowledge about the origin of the coffee and the use of fair trade practices by the producer. This failure is also associated with improper preparation of coffee, with particular emphasis on the correct water temperature and the right dose of coffee.

The respondents mainly choose instant coffee, ground roasted coffee, and roasted coffee beans. Few people choose low-acid or decaffeinated coffee, which may indicate that consumers drink coffee for its flavor and also for the stimulating effect of caffeine, and also that health aspects are not important to them.

The conducted cluster analysis allowed for the identification of three groups (clusters) of respondents drinking coffee. They were classified as “Neutral coffee drinkers” (1), “Ad hoc coffee drinkers” (2), and “Non-specific coffee drinkers” (3). Cluster 1 were people drinking coffee mainly out of habit, not overly concerned with the type or method of preparation. They were mainly women aged 31–40, living in medium-sized cities, and drinking coffee from an espresso machine, which may indicate a preference for the stimulating properties of coffee. Consumers representing the second cluster drink coffee occasionally and, at the same time, are more likely to consume different types of coffee, and prepare it with different methods. They were young people with higher education, living in large cities, treating coffee as a lifestyle, consuming it mainly outside the home, but with little frequency. The third cluster includes the remaining respondents who cannot be characterized clearly.

To conclude, it can be stated that the Polish coffee consumer prefers conventional methods of brewing coffee (he/she is a “traditionalist”), but is also open to novelties and the search for new sensory experiences.

The results of the study can be helpful for coffee cafe owners, retailers, and suppliers of coffee, as well as coffee makers sellers, all of whom aim to adapt to changing consumer habits. Identified factors that influence consumers’ coffee choices and methods of coffee preparation, and pointed consumer habits related to coffee consumption may allow to understand the consumer-making process. A consumer segmentation could be helpful to provide marketing activity among proper consumer group, and can be interesting for other populations to cross-cultural comparison.

It would certainly be worth repeating our study after the COVID-19 pandemic is over. It may be an interesting idea to study the impact of remote working mode on the purchase and use of home coffee machines, as well as evaluation the behavior of consumers consuming coffee in cafes. Further research directions may concern coffee cold brewing, especially among consumers who are open to innovation. It would be interesting to investigate the multisensory perception and cross modal relationships of potential coffee consumers.

## Figures and Tables

**Table 1:** Questionnaire structure.

Question	Answer Options
Q1. How often do you drink coffee (one cup of coffee)? Choose the answer that suits you the best (only one option).	(1) once a day; (2) twice a day; (3) three or four times a day; (4) three or four times a week; (5) once a week; (6) three times a month; (7) once a month; (8) never (if respondents choose this answer, they end the questionnaire)
Q2. What kind of coffee do you like usually drink?	(1) roasted coffee beans; (2) grain coffee (from cereals); (3) decaffeinated coffee; (4) ground roasted coffee (coffee powder); (5) flavored coffee; (6) instant coffee; (7) low-acid coffee; (8) coffee beverages
Q3. What brands of coffee do you choose most often?	Please list: Nescafe, Tchibo, Jacobs, Segafredo, Lavazza, MK Café, Maxwell House, Pedros, Woseba, Astra, other (please specify)
Q4. How do you drink your coffee?	(1) black, unsweetened; (2) with sugar; (3) with sugar substitutes (sweetener); (4) with milk; (5) with cream; (6) other (please specify)
Q5. Is the method of preparation of the coffee infusion important to you?	(1) yes; (2) no; (3) sometimes
Q6. How do you brew your coffee?	(1) in a drip coffee maker; (2) flooded with boiling water in a cup or glass; (3) in a French press coffee maker; (4) cold brew method; (5) in a pressure coffee machine; (6) in a moka pot; (7) percolator coffee maker; (8) AeroPress coffee maker; (9) I don’t brew coffee
Q7. How long do you brew your coffee?	(1) <3 min; (2) 3 min; (3) 4 min; (4) 5 min; (5) 6 min; (6) I do not pay attention to the brewing time
Q8. What type of water do you use to prepare the infusion?	(1) tap water from the mains water supply; (2) filtered water; (3) oligocene water; (4) mineral water still
Q9. How many spoons of coffee do you use for brewing (per cup)?	(1) “more or less”; (2) according to the recommendations on the coffee package; (3) other; (4) 1–1.5 teaspoons; (5) 2–2.5 teaspoons; (6) 3–4 teaspoons; (7) 5 teaspoons or more; (8) a measuring cup for a coffee machine, capsules.
Q10. At what temperature do you brew your coffee?	(1) 100 °C; (2) 93 °C; (3) 98 °C; (4) I don’t pay attention to it
Q11. What kind of coffee drink do you prefer?	(1) Americano—a type of coffee drink prepared by diluting an espresso with hot water; (2) espresso—strong coffee brewed in an espresso machine, served in a small cup; (3) cappuccino—espresso with hot, strongly frothed milk (with a fluffy foam); (4) iced coffee; (5) latte—espresso with a lot of hot, gently frothed milk or latte macchiato—hot milk, espresso, and a fluffy foam; (6) caffé mocha—espresso served with hot milk and dark or milk chocolate;(7) other (please specify)
Q12. What do you appreciate the most about the quality of coffee you drink?	(1) color; (2) flavor; (3) aroma; (4) appearance; (5) coffee strength
Q13. When I buy coffee, I usually take into consideration: 13.1: Price; 13.2: Quality; 13.3: Brand; 13.4: Packaging (attractiveness, information); 13.5: Friends’ opinions; 13.6: Presentation on a shelf in a store; 13.7: Promotion; 13.8: Habits; 13.9: Advertisement; 13.10: Flavor (taste and aroma); 13.11: Convenience; 13.12: Health reasons; 13.13: Others (please specify)	Choose a comment for each statement (Likert scale): (1) Definitely do not agree; (2) Do not agree; (3) I tend to disagree; (4) Undecided; (5) I tend to agree; (6) Agree; (7) Definitely agree
Q14. Where do you drink your coffee? Choose the answers that suits you the best.	(1) at home; (2) in canteens; (3) in a café; (4) at friends’, family members’ homes; (5) at work
Sociodemographic data: Gender:	Choose the right answer: (1): women; (2): men;
Age:	(1): 18–25 years old; (2): 26–30 years old; (3): 31–40 years old;(4): 41–50 years old; (5): 51–65 years old
Education:	(1): vocational or primary school; (2): secondary school; (3): higher education (university);
Dwelling place:	(1): city over 100,000 inhabitants; (2): city between 50,000–100,000 inhabitants; (3): city between 10,000–50,000 inhabitants; (4): city below 10,000 inhabitants and village;

**Table 2:** Characteristics of the surveyed sample of respondents.

Population Features	Group	Number of Respondents (*n*)	Percentage of Respondents (%)
Total	--	1500	100.0
Gender	women	1049	69.9
	men	451	30.1
Age	18–25 years old	435	29.0
	26–30 years old	239	15.9
	31–40 years old	258	17.2
	41–50 years old	312	20.8
	51–65 years old	256	17.1
Education	vocational or primary school	165	11.0
	secondary school	605	40.3
	higher education (university)	730	48.7
Dwelling place	city over 100,000 inhabitants	646	43.1
	city between 50,000–100,000 inhabitants)	212	14.1
	city between 10,000–50,000 inhabitants	396	26.4
	city below 10,000 inhabitants and village	246	16.4

**Table 3:** Places of respondents drink coffee.

Place	Response		*p*-Value *			
	Number	Percentage	Gender	Age	Education	Dwelling Place
at home	1432	95.5	NS	NS	NS	0.024
in canteens	687	45.8	NS	0.000	NS	0.000
in a café	922	61.5	0.006	0.000	0.0006	0.000
at friends’, family members’ homes	994	66.3	0.039	0.000	NS	NS
at work	1196	79.7	NS	0.002	0.000	NS

* NS—no significant, *p* < 0.05

**Table 4:** Factors affecting coffee purchasing.

Factors	The Importance of the Factor *			
	Median Me	Quartile Deviation Q	Q25	Q75
Price	5	±1	4	6
Quality	6	±1	5	7
Brand	5	±1	4	6
Packaging (attractiveness, information)	4	±1	3	5
Friends’ opinion	5	±1	4	6
Presentation on a shelf in a store	4	±1	2	4
Promotion	4	±0.5	4	5
Habits	5.5	±1	4	6
Advertisement	4	±1	2	4
Flavor (taste and aroma)	6	±1.5	4	7
Convenience	4	±0.5	4	5
Health reasons	4	±1	3	5
Others (origin, acidity, strength, degree of roasted)	5	±1	4	6

* Likert scale: (1): Definitely do not agree; (2): Do not agree; (3): I tend to disagree; (4): Undecided; (5): I tend to agree; (6): Agree; (7): Definitely agree; Q25—lower quartile, Q75—upper quartile; Q = (Q75−Q25)/2.

**Table 5:** Preparation methods and types of coffee beverages by respondents.

Brewing Method	Respondents		Type of Coffee Beverages	Respondents	
	*n*	%		*n*	%
in a drip coffee maker	922	61.5	Americano	771	51.4
flooded with boiling water in a cup or glass	1345	89.7	espresso/double espresso	1359	90.6
in French press coffee maker	231	15.4	cappuccino	1262	84.1
cold brew method	193	12.9	iced coffee	1140	76.0
in a pressure coffee machine	1165	77.7	latte/latte macchiato	1119	74.6
in a moka pot	183	12.2	caffé mocha	779	51.9
percolator coffee maker	98	6.5	other	140	9.3
AeroPress coffee maker	186	12.4	-	-	-

Multiple choice question.

**Table 6:** Respondents’ preferences for the preparation of coffee infusion.

Preferences	Respondents		Preferences	Respondents	
	*n*	%		*n*	%
Water Used to Brewing			Brewing Water Temperature		
tap water from the mains water supply	971	64.7	100 °C	748	49.9
Oligocene water	78	5.2	93 °C	269	17.9
mineral water still	169	11.3	98 °C	453	30.2
filtered water	654	43.6	I don’t pay attention to it	30	2.0
The amount of coffee for 1 cup			Preferred coffee brewing time		
“more or less”	312	20.8	<3 min	226	15.1
acc. to the recommendations on the coffee package	85	5.7	3 min	267	17.8
1–1.5 teaspoons	360	24.0	4 min	114	7.6
2–2.5 teaspoons	455	30.3	5 min	82	5.5
3–4 teaspoons	59	3.9	6 min	30	2.0
5 teaspoons or more	14	0.9	I don’t pay attention to the brewing time	781	52.1
a measuring cup for a coffee machine, capsules	172	13.8			
other	43	2.9	-	-	-

## Data Availability

The data presented in this article is available on reasonable request, from the corresponding author.
